# Relationship between Antibiotic Susceptibility and Genotype in *Mycobacterium abscessus* Clinical Isolates

**DOI:** 10.3389/fmicb.2017.01739

**Published:** 2017-09-14

**Authors:** Bing Li, Shiyi Yang, Haiqing Chu, Zhemin Zhang, Weijia Liu, Liulin Luo, Wei Ma, Xiaogang Xu

**Affiliations:** ^1^Department of Respiratory Medicine, Shanghai Pulmonary Hospital, School of Medicine, Tongji University Shanghai, China; ^2^School of Medicine, Tongji University Shanghai, China; ^3^Shanghai Key Laboratory of Tuberculosis, Shanghai Pulmonary Hospital, School of Medicine, Tongji University Shanghai, China; ^4^Department of Clinical Laboratory Medicine, Shanghai Pulmonary Hospital, School of Medicine, Tongji University Shanghai, China; ^5^State Key Laboratory of Microbial Metabolism, School of Life Sciences and Biotechnology, Shanghai Jiao Tong University Shanghai, China; ^6^Institute of Antibiotics, Huashan Hospital, Fudan University Shanghai, China

**Keywords:** resistance, clarithromycin, genotype, *Mycobacterium abscessus*, microorganism

## Abstract

This study aimed to determine the antibiotic susceptibility and resistance related genotypes of *Mycobacterium abscessus*. One hundred sixty-two clinical isolates were collected. Genomic data were obtained by whole genome sequencing. Single nucleotide polymorphism (SNP) analysis was conducted using the NCBI GenBank database and BLAST algorithm. The following genes were of interest: *erm(41), rrl* and *rrs*. *Erm(41)* was further divided into 3 sequevars: *erm(41)C28, erm(41)T28*, and M type [*erm(41)* with deletions in nucleotides 64 and 65, or 159 through 432]. Antibiotic susceptibility was assessed at 3 days (early reading time, ERT) and 14 days (late reading time, LRT) after clarithromycin (CLA) treatment. Three patterns of CLA resistance were observed. (1) Fifty-five (acquired resistance) isolates [45 *erm(41)T28*, 1 *erm(41)C28* and 9 M type] exhibited MIC ≥8 mg/L at ERT; among these isolates, 10 had an *rrl* 2058/2059 mutation. (2) Sixty-two *subsp. abscessus* and 2 *subsp. massiliense* (induced resistance) isolates exhibited MIC ≤4 mg/L at ERT, but ≥8 mg/L at LRT. (3) Forty-three (sensitive and intermediate) isolates [14 *erm(41)C28*, 1 *erm(41)T28*, and 28 M type] exhibited MIC ≤4 mg/L at both ERT and LRT. No *rrs* 1408 mutation or other meaningful SNP was found in 3 amikacin-resistant isolates. No correlation was found between *rrl, erm(41)* or *rrs* and susceptibility to the 8 other antibiotics tested. The *rrl* and *erm(41)* genotypes could predict the CLA resistance of *M. abscessus* clinical isolates. China has a large number of CLA-resistant *M. abscessus* isolates with *erm(41)T28* sequevar. Treatment of *M. abscessus* infections should be based upon a comprehensive consideration of factors that include genotype and geographic location.

## Introduction

The incidence of non-tuberculous mycobacteria (NTM) infections has increased rapidly over the past decade coincidentally with an increase in the number of immune dysfunction cases, as well as the number of endoscopies, surgeries, transplantations and other invasive operations (Griffith et al., 2007; Lai et al., 2010; Hoefsloot et al., 2013; Marras et al., 2013; Brode et al., 2014; Prevots and Marras, 2015). Among these, *M. abscessus* infections are particularly challenging. *M. abscessus* is one of the most antibiotic-resistant pathogens known (Nessar et al., 2012). It is resistant to most first-line antibiotics, and naturally resistant to antitubercular agents. Consequently, even treatment with a combination of antibiotics over a long period of time is often ineffective (Jarand et al., 2011).

The 2007 American Thoracic Society guidelines recommended the inclusion of clarithromycin (CLA) in combination therapy for *M. abscessus* infections (Griffith et al., 2007). In recent years, however, CLA efficacy was found closely related to bacterial genotype (Wallace et al., 1996; Nash et al., 2009; Bastian et al., 2011; Lee et al., 2014; Brown-Elliott et al., 2015; Rubio et al., 2015). Two kinds of genotype-related CLA resistance patterns have been confirmed. (1) A point mutation in the 2058/2059 (*E. coli* numbering) locus of the 23S rRNA (*rrl*) gene confers acquired resistance. (2) An intact *erm(41)* gene, which exhibits a T/C polymorphism at the 28th nucleotide, confers inducible resistance to CLA when thymidine is the nucleotide [*erm(41)T28*]. The CLA-induced *erm(41)T28* gene product methylates ribosomal 23S mRNA, preventing CLA binding to the ribosome (Bastian et al., 2011). Additionally, there are two sensitivity patterns: (1) When cytidine is the 28th nucleotide [*erm(41)C28*], there is a loss of both adenine methylating function and CLA resistance. (2) Deletion of *erm(41)* nucleotides 64 and 65, and deletion of *erm(41)* nucleotides 159–432; both deletions result in the loss of gene function (M type) (Nash et al., 2009). *M. abscessus* genotype and CLA phenotype are correlated. To determine the CLA resistance phenotype (i.e., sensitive, acquired resistance or induced resistance), it is necessary to conduct *in vitro* susceptibility testing on day 3 (early reading time, ERT) and day 14 (late reading time, LRT) after CLA treatment.

Amikacin is also a recommended, primary therapeutic agent for treatment of *M. abscessus* infections. One underlying mechanism for amikacin resistance seems to be an A to G mutation in nucleotide 1408 of the *rrs* gene (Nessar et al., 2011). Taken together, these patterns indicate that *M. abscess*us antibiotic susceptibility studies should focus on the following genotypes: (1) *erm(41)* [including *erm(41)T28, erm(41)C28* and M type], (2) the 2058/2059 *rrl* gene mutation, and (3) the 1408 *rrs* mutation.

Identification of the genotype of infectious *M. abscess*us is instructive when selecting antimicrobial agents for treatment. Recently, Mougari and co-workers correlated the results of standardized antibiotic susceptibility testing with *M. abscessus* genotyping (Mougari et al., 2016). Their analyses confirmed that *rrl, erm(41)* and *rrs* genotypes accounted for and predicted the resistance to CLA and amikacin exhibited by clinical *M. abscessus* isolates in France. Therefore, therapeutic approaches to treating *M. abscessus* infections should consider the genotype involved although the character of the etiologic agent is frequently not known in such detail. China has a large number of patients with *M. abscessus* infection. But so far, there is no systematic study on the relationship between susceptibility and genotype of *M. abscessus* clinical isolates in China. Recent articles have only explored the relationship between susceptibility and subtype (Pang et al., 2015), or just mentioned that the *erm(41)* genotype was related to clarithromycin resistance after 14 days' incubation, without detailed genetic information or susceptibility test results (Nie et al., 2015). Moreover, the genotype and antibiotic susceptibility of *M. abscessus* may vary worldwide (Harada et al., 2012; Lee et al., 2014; Brown-Elliott et al., 2015; Nie et al., 2015; Pang et al., 2015; Rubio et al., 2015; Cowman et al., 2016; Garcia de Carvalho et al., 2016; Mougari et al., 2016). The study described herein was undertaken to establish the relationship between antibiotic susceptibility and *M. abscessus* genotype in China, and to compare it to that found in other countries.

## Materials and methods

### Bacteria

One hundred sixty-two *M. abscessus* isolates were collected at Shanghai Pulmonary Hospital. Shanghai Pulmonary Hospital is one of the designated treatment centers for Chinese tuberculosis and NTM infections, attracting NTM cases from across the country. Clinical strains were isolated from the sputum specimens of suspected tuberculosis patients in Shanghai (39 isolates), Jiangsu (21 isolates), Zhejiang (22 isolates), Jiangxi (19 isolates), Anhui (15 isolates), Hunan (10 isolates), Shandong (10 isolates), Fujian (6 isolates), Henan (4 isolates), Gansu (3 isolates), Liaoning (3 isolates), Jilin (3 isolates), Hubei (2 isolates), Heilongjiang (2 isolates), Xinjiang (2 isolates), and Sichuan (1 isolate) Provinces of China. 42 clinical strains were isolated in 2012, 38 in 2013, 38 in 2014, and 44 in 2015. Among them, one hundred and eight isolates were from the sputum specimens, and 54 were from the lavage fluid samples. No cystic fibrosis cases were included. All isolates were identified as NTM by growth in MGIT960 culture medium and the *p*-nitrobenzoic acid test. *M. abscessus* isolates were further characterized by sequencing the *rpoB* gene. This study was carried out in accordance with the recommendations of the guidelines of Medical Ethics of Scientific Research of Tongji University, Ethics Committee of Tongji University and Shanghai Pulmonary Hospital with written informed consent from all subjects. All subjects gave written informed consent in accordance with the Declaration of Helsinki.

### DNA extraction, library construction, illumina HiSeq sequencing and genome assembly

#### DNA extraction

DNA extraction was performed as we described previously with slight modification (Somerville et al., 2005; Luo et al., 2016). Ten microliters of bacteria were transferred to a microcentrifuge tube containing TE buffer and glass beads. After vortexing, an aliquot was incubated with lysozyme overnight at 37°C. Subsequently, sodium dodecyl sulfate and proteinase K were added, and the incubation was continued at 65°C for 20 min. Then, 10% N-acetyl-N,N,N-trimethyl ammonium bromide and 0.7 M NaCl were added followed by 0.5 M NaCl, and the mixture was incubated for 10 min at 65°C. Afterwards, chloroform-isoamylalcohol (24:1) was added, and the tube was centrifuged at 13,000 rpm. The DNA remaining in the aqueous phase was precipitated with isopropanol and collected by centrifuged at 13,000 rpm. The precipitate was washed with ethanol and dissolved in TE buffer. Genomic DNA was quantified using a NanoDrop 2000 Spectrophotometer (Thermo Fisher Scientific Inc., West Palm Beach, FL, USA). High-quality DNA samples (OD260/280 = 1.8–2.0) were used to construct 350–450 bp fragment libraries.

#### Library construction and illumina HiSeq sequencing

Purified genomic DNA derived from each strain was used to construct a sequencing library. Paired-end libraries with insert sizes of ~400 bp were prepared following Illumina's standard genomic DNA library preparation protocol (Illumina, USA). Purified genomic DNA was sheared into smaller fragments using a Covaris focused ultrasonicator (Thermo Fisher Scientific, Waltham, MA, USA); blunt ends were generated with T4 DNA polymerase. After adding an “A” base to the 3' ends, adapters were ligated to each end of the blunt phosphorylated DNA fragments. The fragments were purified by gel-electrophoresis, then amplified by PCR. An index tag was introduced into the adapter during PCR and a library quality test was performed. Finally, the qualified Illumina paired-end library was used for Illumina Hiseq sequencing (150 bp^*^2).

#### Genome assembly

Prior to using the default parameters of the SPAdes software (Version v.3.6.0, http://bioinf.spbau.ru/en/spades) to assemble the genome draft (Bankevich et al., 2012), SPAdes was combined with BayesHammer to adjust the bases and to correct any sequence assembly errors and incomplete insertions (Nikolenko et al., 2013). The result of the assembly was evaluated using QUAST (Version v.2.3, http://quast.bioinf.spbau.ru/; Gurevich et al., 2013).

#### Nucleotide sequence accession number

The accession numbers for the 162 *M. abscessus* isolates sequenced in this study are available at DDBJ/ENA/GenBank under the bioproject PRJNA 398137.

### SNP analysis

The NCBI Nucleotide blast program was used for SNP analysis. *M. abscess*us standard strain ATCC19977 (NC_010397.1) was selected as the reference strain for *rrl* and *erm(41)T28*; CR5701 (HQ127366.1) was selected as the reference strain for *erm(41)C28*; and CCUG48898 (AP014547.1) was selected as the reference strain for M type.

### Susceptibility testing

Antibiotic susceptibility was determined by the microdilution method. Sulfonamides, moxifloxacin, cefoxitin, amikacin, doxycycline, tigecycline, clarithromycin, linezolid, imipenem, and tobramycin, which are among the most common antibiotics used to treat *M. abscessus* infections, were tested (TREK Diagnostic Systems, USA). The susceptibility was assessed at 3 days (early reading time, ERT) and 14 days (late reading time, LRT) after *M. abscessus* complex group identification. Sensitive isolates exhibited MIC ≤2 mg/L at both ERT and LRT; intermediate isolates exhibited MIC = 4 mg/L at both ERT and LRT; acquired resistance isolates exhibited MIC ≥8 mg/L at ERT; induced resistance isolates exhibited MIC ≤4 mg/L at ERT, but ≥8 mg/L at LRT. Antibiotics susceptible and resistant breakpoints were interpreted according to Clinical Laboratory Standards Institute (CLSI)-M24-A2 (Clinical and Laboratory Standards Institute, 2011). *Staphylococcus aureus* (ATCC29213; American Type Culture Collection, Manassas, VA, USA) was used as the control reference strain.

## Results

One hundred sixty-two *M. abscessus* isolates were collected, including 123 subsp. *abscessus* and 39 subsp. *massiliense*. *M. abscessus* subsp. *bolletii* was not considered in this study due to its general absence in China. One hundred eight *erm(41)T28* sequevar and 15 *erm(41)C28* sequevar were among subsp. *abscessus*. The susceptibilities of these *erm(41)* sequevars to the 10 antibiotics tested were shown in Table 1. While a relatively large percentage of the *erm(41)T28* sequevar exhibited both acquired and inducible resistance to CLA, only 3 isolates exhibited resistance to amikacin. Neither an *rrs* 1408 mutation nor any other meaningful SNP was found in these 3 isolates. No correlation was observed between genotype and susceptibility to the 8 other antibiotics tested. The MIC50 and MIC90 values for all 10 antibiotics were shown in the Supplementary Table 1. The phylogenetic tree of 162 *M. abscessus* was also constructed, with information of genotypes and clarithromycin/amikacin-resistant types for each strain labeled (Supplementary Figure 1).

**Table 1:** Antibiotic resistance of all *M. abscessus* isolates^a^.

**Antibiotic**	**MIC (mg/mL)**			**Number of resistant isolates (%)**		
	**Sensitive**	**Intermediate**	**Resistant**	***erm(41)C28* (*n* = 15)**	***erm(41)T28* (*n* = 108)**	**M type (*n* = 39)**
CLA (resistant before induction)	≤2	4	≥8	1(6.7)	45(41.7)	9(23.1)
CLA (resistant after induction)	≤2	4	≥8	1(6.7)	107(99.1)	11(28.2)
AMI (amikacin)	≤16	32	≥64	1(6.7)	2(1.9)	0(0.0)
SXT (sulfonamides)	≤2	nrb^b^	≥4	7(46.7)	61(56.5)	25(64.1)
MXF(moxifloxacin)	≤1	2	≥4	15(100.0)	107(99.1)	38(97.4)
FOX (cefoxitin)	≤16	32–64	≥128	12(80.0)	67(62.0)	27(69.2)
DOX (doxycycline)	≤1	2–4	≥8	15(100.0)	107(99.1)	39(100)
TGC (tigecycline)	≤1	nrb	nrb	nd^c^	nd	nd
LZD (linezolid)	≤8	16	≥32	8(53.3)	50(46.3)	15(38.5)
IMI (imipenem)	≤4	8–16	≥32	15(100.0)	103(95.4)	38(97.4)
TOB (tobramycin)	≤2	4	≥8	15(100.0)	94(87.0)	38(97.4)

a *The erm(41) sequevar-dependent resistance of 162 M. abscessus isolates to the antibiotic indicated was determined by the microdilution method. The incubation time was 3 days (before induction) and 14 days (after induction) for CLA, and 3 days for the other antibiotics listed*.

b *no recommended breakpoint*.

c *no data*.

The effects of *M. abscessus* subtype, *erm(41)* sequevar and incubation time on the MIC values of CLA were determined (Table 2). There were 66 CLA-sensitive (MIC ≤2 mg/mL) and 11 CLA-intermediate (MIC = 4 mg/mL), *M. abscessus* subsp. *abscessus* isolates [14 *erm(41)C28* and 63 *erm(41)T28* with no *rrl* 2058/2059 mutation] accessed at ERT. Forty-six acquired resistance isolates (MIC >8 mg/mL) accessed at the same time included 45 *erm(41)T28*, 2 with an *rrl* 2058/2059 mutation, and 1 *erm(41)C28* isolate with an *rrl* 2058/2059 mutation. There were 29 CLA-sensitive and 1 CLA-intermediate subsp. *massiliense* isolates with no *rrl* 2058/2059 mutation, and 9 acquired resistance isolates, 7 of which had an *rrl* 2058/2059 mutation. Analysis of the *rrl* mutation pattern revealed 7 isolates with a 2058 mutation (5 isolates exhibiting a A to C mutation and 2 isolates exhibiting a A to G mutation); 2 isolates exhibited a 2059, A to C mutation. The remaining two resistant subsp. *massiliense* isolates, which exhibited no *rrl* 2058/2059 mutation, were analyzed to determine the presence of an 18 bp insertion mutation in the *rpIV* gene (encoding 50S ribosomal protein L22), no such insertion was found (data not shown).

**Table 2:** The effects of *M. abscessus* subtype, *erm(41)* sequevar and incubation time on the MIC values of CLA.

**CLA MIC (mg/L)**	**All subtypes (*n* = 162)**		**subsp. *abscessus* (*n* = 123)**				**subsp. *massiliense* (*n* = 39)**	
	**All sequevars (*n* = 162)**		***erm(41)T28* (*n* = 108)**		***erm(41)C28* (*n* = 15)**		**M type(*n* = 39)**	
	**ERT**	**LRT**	**ERT**	**LRT**	**ERT**	**LRT**	**ERT**	**LRT**
0.06	7	2	0	0	0	0	7	2
0.12	9	13	0	0	4	1	5	12
0.16	2	1	0	0	1	0	1	1
0.25	9	6	3	0	0	3	6	3
0.5	23	11	9	1	6	4	8	6
1	24	9	19	0	3	5	2	4
2	21	1	21	0	0	1	0	0
4	12	0	11	0	0	0	1	0
8	14	1	14	0	0	0	0	1
16	41	118	31	107	1	1	9	10
≤2(^*rrl* mut^)	95^(0)^	43	52^(0)^	1	14^(0)^	14	29^(0)^	28
≤4(^*rrl* mut^)	107^(0)^	43	63^(0)^	1	14^(0)^	14	30^(0)^	28
≥8(^*rrl* mut^)	55^(10)^	119	45^(2)^	107	1^(1)^	1	9^(7)^	11

*(rrl mut) Number of stains with rrl 2058/2059 mutation*.

There were 15 CLA-sensitive subsp. *abscessus* isolates [14 *erm(41)C28* and 1 *erm(41)T28*] and 108 (inducible) resistant isolates [1 *erm(41)C28*] assessed at LRT. Twenty-eight CLA-sensitive and 11 CLA-resistant subsp. *massiliense* were also determined at LRT (Table 2).

Sixty-two subsp. *abscessus* isolates, all of which were *erm(41)T28*, and 2 subsp. *massiliense* exhibited inducible resistance to CLA assessed at LRT. The correlation between CLA susceptibility and *M. abscessus* genotypes isolated in China was shown in Table 3. The same correlation for *M. abscessus* isolated in Spain, France and the US, gleaned from data reported by other investigators (Brown-Elliott et al., 2015; Rubio et al., 2015; Mougari et al., 2016), was shown for comparison purposes (Supplementary Table 2).

**Table 3:** Distribution and CLA susceptibility patterns among *M. abscessus* genotypes^a^.

**Country**	**Total number**	***Erm(41)***			***rrl* mut**	***rrl* mut/ERT resistant**	**Induced resistance^b^**			**LRT susceptible and intermediate**		
		**T28**	**C28**	**M**			**T28**	**C28**	**M**	**T28**	**C28**	**M**
China	162	108	15	39	10	18.2% (10/55)	96.9% (62/64)	0	3.1% (2/64)	2.3 (1/43)	32.6% (14/43)	65.1% (28/43)

a *Genotypes include erm(41)C28, erm(41)T28, M type, and rrl 2058/2059 mutation (rrl mut)*.

b *Sensitive/intermediate at ERT, but resistant at LRT*.

## Discussion

This study was the first undertaken to correlate antibiotic susceptibility with the genotypes of *M. abscessus* isolated in China. The proportion of *M. abscessus* isolates that acquired resistance to CLA (ERT assessment) was relatively high (33.95%, 55/162) compared to other countries: Japan, 12.79% (11/86); Korea, 15.84% (64/404); UK, 6.54% (35/535); France, 9.09% (15/165); US, 2.51% (9/358); and Brazil, 5.55% (2/36). The countries included in this comparison consisted of those where *M. abscessus* infections were most frequently studied and, consequently, were the largest sources of available data over the last 5 years (Harada et al., 2012; Lee et al., 2014; Brown-Elliott et al., 2015; Nie et al., 2015; Pang et al., 2015; Rubio et al., 2015; Cowman et al., 2016; Garcia de Carvalho et al., 2016; Mougari et al., 2016). Undoubtedly, uneven economic development, and differences in the production, management and clinical use of antibiotics contribute to this disparity in rates of CLA resistance observed among countries.

Further analysis indicated that only 3 of the ERT resistant subsp. *abscessus* isolates had an *rrl* 2058/2059 mutation. The *rrl* SNP distribution of the remaining 43 isolates was scattered and irregular, but all isolates belong to the *erm(41)T28* sequevar. As *erm(41)T28* is an inducible resistance genotype, we speculated that resistance may have occurred in a large portion of these isolates as a consequence of contact with macrolide agents such as CLA prior to isolation. This speculation is based upon the following considerations. According to Chinese community-acquired pneumonia (CAP) guidelines, macrolides were once the primary agents of choice to treat a high proportion of mycoplasma pneumoniae and mixed, atypical pathogens infections (Xu et al., 2007). Consequently, many Chinese CAP patients including patients enrolled in the present study may have a history of macrolide use. Additionally, China has a large number of patients with bronchiectasis, which is linked closely with incidences of NTM infection (Chu et al., 2014). Notably, macrolides are widely recommended for long-term treatment of bronchiectasis patients (Fan and Xu, 2014). Therefore, prior use in treating bronchiectasis may also be a factor contributing to the high rate of macrolide resistance exhibited by pathogenic *M. abscessus* isolated in China. Confirming this speculation will require detailed histories of the patients and their antibiotic use, as well as analyses of the *erm(41)* expression level and 23S rRNA methylation status of the infectious organism. This work is ongoing in our laboratory.

There were 9 CLA-resistant subsp. *massiliense* isolates assessed at ERT, only 7 exhibited an *rrl* 2058/2059 mutation. Since subsp. *massiliense* possesses an incomplete/nonfunctional *erm(41)* (M type), the mechanism underlying resistance of the 2 M type *erm(41)* strains with no *rrl* 2058/2059 mutation remains to be determined. However, it has been reported that M type isolates will ultimately acquire resistance provided long-term CLA exposure; the underlying mechanism(s) appears to be a change in 50S ribosomal subunit structure (Mougari et al., 2017). Following exposure, 97.5% (39/40) M type strains exhibited an *rrl* 2058/2059 mutation; 2.5% (1/40) had an 18 bp insertion mutation in the *rpIV* gene, which encodes the L22 ribosomal protein. In the present study, CLA resistance in subsp. *massiliense* was principally due to an *rrl* 2058/2059 mutation, which occurred in 77.8% (7/9) of isolates; no *rpIV* mutation was found in the 2 resistant isolates that remained.

To further understand the relationship between *M*. *abscessus* genotype and CLA susceptibility typical of different geographic regions, regions with detailed genotypes and CLA susceptibility data were selected and the data compared with those obtained in China (Table 3 and Supplementary Table 2) (Brown-Elliott et al., 2015; Rubio et al., 2015; Mougari et al., 2016). Sixty to hundred percent of the acquired, CLA-resistant *M. abscessus* isolated in Europe and the US, but only 18.2% in China, expressed an *rrl* mutation. Most acquired, CLA-resistant isolates in China might be macrolide-induced *erm(41)T28* strains. The vast majority (96.9–100%) of the induced CLA-resistant strains were *erm(41)T28*. Only 2 M type strains in our study appeared induced resistance, the mechanism is unknown. One CLA-resistant *erm(41)C28* was isolated. Studies suggest, that CLA resistance can be induced in *erm(41)C28* sequevar by long-term exposure to higher CLA concentrations (Maurer et al., 2012; Mougari et al., 2017). This might require a mutation in the *rrl* gene, which is consistent with our findings.

CLA-sensitive isolates assessed at LRT belonged mainly to the *erm(41)C28* and M type sequevars; one, however, belonged to *erm(41)T28*. The mechanism(s) underlying the CLA sensitivity of the *erm(41)T28* isolates remains to be elucidated.

*M. abscessus* isolates that belong to the *erm(41)C28* or M type sequevar, which do not express an *rrl* 2058/2059 mutation, are probably susceptibility to CLA treatment. In the case of patients infected with *erm(41)T28* sequevar, treatment options should consider geographic location and patient medical history; response to treatment and recovery should be closely monitored.

Only 3 (1.9%, 3/162) of the *M. abscessus* isolates recovered in China exhibited resistance to amikacin, neither an *rrs* 1408 mutation nor any other meaningful SNP was determined. Consequently, amikacin appears to have a higher efficiency rate than CLA in treating *M. abscessus* infections in China. The requirement for frequent and long-term intramuscular or intravenous administration undoubtedly impacts negatively on the clinical use of amikacin. Recently, Olivier et al. reported the results of a phase II clinical trial in which long-term liposomal amikacin inhalation combined with traditional therapy improved the outcomes of the sputum culture and 6-min walk test in NTM infected patients (Olivier et al., 2016). These findings suggest that amikacin may be a good, alternative for *M. abscessus* treatment.

Our results are consistent with those obtained in other studies with the exception that most *M. abscessus* isolates obtained in China were resistant to imipenem and cefoxitin (Griffith et al., 2007; Mougari et al., 2016). Therefore, a better treatment for *M. abscessus* infections in China might be CLA combined with amikacin or linezolid, while European and American researchers recommended the use of cefoxitin as an alternative agent (Griffith et al., 2007; Harada et al., 2012; Nie et al., 2015; Pang et al., 2015; Cowman et al., 2016; Mougari et al., 2016). Imipenem should not be considered for early treatment in China. No resistance-related genotypes for the other 8 antibiotics tested were found.

## Conclusion

The *rrl* and *erm(41)* genotypes are predictive of the resistance of *M. abscessus* clinical isolates to CLA. China has a large number of CLA-resistant *M. abscessus* isolates with *erm(41)T28* sequevar. Treatment of *M. abscessus* infections should be based upon a comprehensive consideration of factors that include genotype and geographic location.

## Author contributions

HC, BL, SY, and ZZ, conceived and designed the work; WL, SY and LL collected the bacterial isolates and compiled the data; WM, BL and LL analyzed and interpreted the data; HC, ZZ and XX revised the manuscript for important intellectual content. All authors approve the final draft of this manuscript. HC is accountable for all aspects of the work.

### Conflict of interest statement

The authors declare that the research was conducted in the absence of any commercial or financial relationships that could be construed as a potential conflict of interest. The reviewer MY declared a past co-authorship with one of the authors XX to the handling Editor.

## References

[B1] Bankevich A. Nurk S. Antipov D. Gurevich A. A. Dvorkin M. Kulikov A. S.. (2012). SPAdes: a new genome assembly algorithm and its applications to single-cell sequencing. J. Comput. Biol. 19, 455–477. 10.1089/cmb.2012.0021 22506599 PMC3342519

[B2] Bastian S. Veziris N. Roux A. L. Brossier F. Gaillard J. L. Jarlier V.. (2011). Assessment of clarithromycin susceptibility in strains belonging to the *Mycobacterium abscessus* group by erm(41) and rrl sequencing. Antimicrob. Agents Chemother. 55, 775–781. 10.1128/AAC.00861-10 21135185 PMC3028756

[B3] Brode S. K. Daley C. L. Marras T. K. (2014). The epidemiologic relationship between tuberculosis and non-tuberculous mycobacterial disease: a systematic review. Int. J. Tuberc. Lung Dis. 18, 1370–1377. 10.5588/ijtld.14.0120 25299873

[B4] Brown-Elliott B. A. Vasireddy S. Vasireddy R. Iakhiaeva E. Howard S. T. Nash K.. (2015). Utility of sequencing the erm(41) gene in isolates of *Mycobacterium abscessus* subsp. Abscessus with low and intermediate clarithromycin MICs. J. Clin. Microbiol. 53, 1211–1215. 10.1128/JCM.02950-14 25653399 PMC4365201

[B5] Chu H. Zhao L. Xiao H. Zhang Z. Zhang J. Gui T.. (2014). Prevalence of nontuberculous mycobacteria in patients with bronchiectasis: a meta-analysis. Arch. Med. Sci. 10, 661–668. 10.5114/aoms.2014.44857 25276148 PMC4175767

[B6] Clinical and Laboratory Standards Institute (2011). Susceptibility Testing of Mycobacteria, Nocardia, and Other Aerobic Actinomycetes; Approved Standard M24-A2. Wayne, PA: CLSI. 31339680

[B7] Cowman S. Burns K. Benson S. Wilson R. Loebinger M. R. (2016). The antimicrobial susceptibility of non-tuberculous mycobacteria. J. Infect. 72, 324–331. 10.1016/j.jinf.2015.12.007 26723913

[B8] Fan L. C. Xu J. F. (2014). Advantages and drawbacks of long-term macrolide use in the treatment of non-cystic fibrosis bronchiectasis. J. Thorac. Dis. 6, 867–871. 10.3978/j.issn.2072-1439.2014.07.24 25093082 PMC4120182

[B9] Garcia de Carvalho N. F. Sato D. N. Pavan F. R. Ferrazoli L. Chimara E. (2016). Resazurin microtiter assay for clarithromycin susceptibility testing of clinical isolates of *Mycobacterium abscessus* group. J. Clin. Lab. Anal. 30, 751–755. 10.1002/jcla.21933 27169515 PMC6806693

[B10] Griffith D. E. Aksamit T. Brown-Elliott B. A. Catanzaro A. Daley C. Gordin F.. (2007). An official ATS/IDSA statement: diagnosis, treatment, and prevention of nontuberculous mycobacterial diseases. Am. J. Respir. Crit. Care Med. 175, 367–416. 10.1164/rccm.200604-571ST 17277290

[B11] Gurevich A. Saveliev V. Vyahhi N. Tesler G. (2013). QUAST: quality assessment tool for genome assemblies. Bioinformatics 29, 1072–1075. 10.1093/bioinformatics/btt086 23422339 PMC3624806

[B12] Harada T. Akiyama Y. Kurashima A. Nagai H. Tsuyuguchi K. Fujii T.. (2012). Clinical and microbiological differences between *Mycobacterium abscessus* and *Mycobacterium massiliense* lung diseases. J. Clin. Microbiol. 50, 3556–3561. 10.1128/JCM.01175-12 22915613 PMC3486228

[B13] Hoefsloot W. van Ingen J. Andrejak C. Angeby K. Bauriaud R. Bemer P.. (2013). The geographic diversity of nontuberculous mycobacteria isolated from pulmonary samples: an NTM-NET collaborative study. Eur. Respir. J. 42, 1604–1613. 10.1183/09031936.00149212 23598956

[B14] Jarand J. Levin A. Zhang L. Huitt G. Mitchell J. D. Daley C. L. (2011). Clinical and microbiologic outcomes in patients receiving treatment for *Mycobacterium abscessus* pulmonary disease. Clin. Infect. Dis. 52, 565–571. 10.1093/cid/ciq237 21292659

[B15] Lai C. C. Tan C. K. Chou C. H. Hsu H. L. Liao C. H. Huang Y. T.. (2010). Increasing incidence of nontuberculous mycobacteria, Taiwan, 2000-2008. Emerging Infect. Dis. 16, 294–296. 10.3201/eid1602.090675 20113563 PMC2958002

[B16] Lee S. H. Yoo H. K. Kim S. H. Koh W. J. Kim C. K. Park Y. K.. (2014). The drug resistance profile of *Mycobacterium abscessus* group strains from Korea. Ann. Lab. Med. 34, 31–37. 10.3343/alm.2014.34.1.31 24422193 PMC3885770

[B17] Luo L. Liu W. Li B. Li M. Huang D. Jing L.. (2016). Evaluation of matrix-assisted laser desorption ionization-time of flight mass spectrometry for identification of *Mycobacterium abscessus* subspecies according to whole-genome sequencing. J. Clin. Microbiol. 54, 2982–2989. 10.1128/JCM.01151-16 27682129 PMC5121389

[B18] Marras T. K. Mendelson D. Marchand-Austin A. May K. Jamieson F. B. (2013). Pulmonary nontuberculous mycobacterial disease, Ontario, Canada, 1998-2010. Emerg. Infect. Dis. 19, 1889–1891. 10.3201/eid1911.130737 24210012 PMC3837646

[B19] Maurer F. P. Ruegger V. Ritter C. Bloemberg G. V. Bottger E. C. (2012). Acquisition of clarithromycin resistance mutations in the 23S rRNA gene of *Mycobacterium abscessus* in the presence of inducible erm(41). J. Antimicrob. Chemother. 67, 2606–2611. 10.1093/jac/dks279 22833642

[B20] Mougari F. Amarsy R. Veziris N. Bastian S. Brossier F. Bercot B.. (2016). Standardized interpretation of antibiotic susceptibility testing and resistance genotyping for *Mycobacterium abscessus* with regard to subspecies and erm41 sequevar. J. Antimicrob. Chemother. 71, 2208–2212. 10.1093/jac/dkw130 27147307

[B21] Mougari F. Bouziane F. Crockett F. Nessar R. Chau F. Veziris N.. (2017). Selection of resistance to clarithromycin in *Mycobacterium abscessus* subspecies. Antimicrob. Agents Chemother. 61:e00943–16. 10.1128/AAC.00943-16 27799212 PMC5192163

[B22] Nash K. A. Brown-Elliott B. A. Wallace R. J. Jr. (2009). A novel gene, erm(41), confers inducible macrolide resistance to clinical isolates of *Mycobacterium abscessus* but is absent from *Mycobacterium chelonae*. Antimicrob. Agents Chemother. 53, 1367–1376. 10.1128/AAC.01275-08 19171799 PMC2663066

[B23] Nessar R. Cambau E. Reyrat J. M. Murray A. Gicquel B. (2012). *Mycobacterium abscessus*: a new antibiotic nightmare. J. Antimicrob. Chemother. 67, 810–818. 10.1093/jac/dkr578 22290346

[B24] Nessar R. Reyrat J. M. Murray A. Gicquel B. (2011). Genetic analysis of new 16S rRNA mutations conferring aminoglycoside resistance in *Mycobacterium abscessus*. J. Antimicrob. Chemother. 66, 1719–1724. 10.1093/jac/dkr209 21652621 PMC3133489

[B25] Nie W. Duan H. Huang H. Lu Y. Chu N. (2015). Species Identification and clarithromycin susceptibility testing of 278 clinical nontuberculosis mycobacteria isolates. Biomed Res. Int. 2015:506598. 10.1155/2015/506598 26146620 PMC4469756

[B26] Nikolenko S. I. Korobeynikov A. I. Alekseyev M. A. (2013). BayesHammer: bayesian clustering for error correction in single-cell sequencing. BMC Genomics 14(Suppl. 1):S7. 10.1186/1471-2164-14-S1-S7 23368723 PMC3549815

[B27] Olivier K. N. Griffith D. E. Eagle G. McGinnis Ii J. P. Micioni L. Liu K.. (2016). Randomized trial of liposomal amikacin for inhalation in nontuberculous mycobacterial lung disease. Am. J. Respir. Crit. Care Med. 195, 814–823. 10.1164/rccm.201604-0700OC 27748623 PMC5363966

[B28] Pang H. Li G. Zhao X. Liu H. Wan K. Yu P. (2015). Drug susceptibility testing of 31 antimicrobial agents on rapidly growing mycobacteria isolates from china. Biomed Res. Int. 2015:419392. 10.1155/2015/419392 26351633 PMC4550772

[B29] Prevots D. R. Marras T. K. (2015). Epidemiology of human pulmonary infection with nontuberculous mycobacteria: a review. Clin. Chest Med. 36, 13–34. 10.1016/j.ccm.2014.10.002 25676516 PMC4332564

[B30] Rubio M. March F. Garrigo M. Moreno C. Espanol M. Coll P. (2015). Inducible and acquired clarithromycin resistance in the *Mycobacterium abscessus* complex. PLoS ONE 10:e0140166. 10.1371/journal.pone.0140166 26448181 PMC4598034

[B31] Somerville W. Thibert L. Schwartzman K. Behr M. A. (2005). Extraction of *Mycobacterium tuberculosis* DNA: a question of containment. J. Clin. Microbiol. 43, 2996–2997. 10.1128/JCM.43.6.2996-2997.2005 15956443 PMC1151963

[B32] Wallace R. J. Jr. Meier A. Brown B. A. Zhang Y. Sander P. Onyi G. O.. (1996). Genetic basis for clarithromycin resistance among isolates of *Mycobacterium chelonae* and *Mycobacterium abscessus*. Antimicrob. Agents Chemother. 40, 1676–1681. 8807061 10.1128/aac.40.7.1676 PMC163394

[B33] Xu Z. J. Ding K. Huang H. Sun T. Y. Ke H. X. Tong Z. H.. (2007). Questionnaire survey of guideline implementation for community acquired pneumonia with retrospective analysis of the empiric treatment in 490 patients. Zhonghua Jie He He Hu Xi Za Zhi 30, 442–446. 17673019

