# Supplementary material for: Relationship between Antibiotic Susceptibility and Genotype in Mycobacterium abscessus Clinical Isolates
Source: Front Microbiol. 2017 Sep 14;8:1739. doi: 10.3389/fmicb.2017.01739 (PMC5603792; doi:10.3389/fmicb.2017.01739)
Supplement: Supplementary file 1 [file Table1.DOCX]

Supplementary Table 1. Antibiotic MIC50 and MIC90 for the subspecies and erm41 sequevars of *M. abscessus* clinical isolates^a^

|  | *M. abscessus* (n=162) | | | *abscessus*  C28 (n=108) | | *abscessus*  T28 (n=15) | | *massiliense* M type (n=39) | |  | | |
| --- | --- | --- | --- | --- | --- | --- | --- | --- | --- | --- | --- | --- |
| Antibiotic | MIC50 | MIC90 | MIC range (mg/L) | MIC50 | MIC90 | MIC50 | MIC90 | MIC50 | MIC90 | |  |  |
| Clarithromycin | 2 | 16 | 0.06 - 16 | 0.5 | 1 | 4 | 16 | 0.5 | 16 | | |  |
| Amikacin | 8 | 16 | 2 - 64 | 16 | 16 | 8 | 16 | 8 | 16 | | |  |
| Tobramycin | 16 | 16 | 2 - 16 | 8 | 16 | 8 | 16 | 16 | 16 | | |  |
| Cefoxitin | 128 | 128 | 32 - 128 | 128 | 128 | 128 | 128 | 128 | 128 | | |  |
| Imipenem | 64 | 64 | 16 - 64 | 64 | 64 | 64 | 64 | 64 | 64 | | |  |
| Linezolid | 16 | 32 | 1 - 32 | 32 | 32 | 16 | 32 | 16 | 32 | | |  |
| Tigecycline | 1 | 4 | 0.25 - 4 | 1 | 4 | 1 | 2 | 1 | 4 | | |  |
| Doxycycline | 16 | 16 | 4 - 16 | 16 | 16 | 16 | 16 | 16 | 16 | | |  |
| Moxifloxacin | 8 | 8 | 0.25 - 8 | 8 | 8 | 8 | 8 | 8 | 8 | | |  |
| Sulfonamides | 4/76^b^ | 8/152 | 0.25/4.75~8/152 | 2/38 | 8/152 | 4/76 | 8/152 | 4/76 | 8/152 | | |  |

^a^The MIC50 and MIC90 (mg/L) of the 10 antibiotics listed were determined at ERT for 162 clinical *M. abscessus* isolates divided into subspecies and *erm(41)* sequevars as indicated.

^b^The breakpoints for sulfamethoxazole and trimethoprim comprising sulfonamides.

Supplementary Table 2. Distribution and CLA susceptibility patterns among *Mycobacterium abscessus* genotypes^a^

| Country | Total number | *Erm(41)* | | | *rrl* mut | *rrl* mut /ERT resistant | Induced resistance^b^ | | | LRT susceptible and intermediate | | | |
| --- | --- | --- | --- | --- | --- | --- | --- | --- | --- | --- | --- | --- | --- |
|  |  | T28 | C28 | M |  |  | T28 | C28 | M | | T28 | C28 | M |
| China | 162 | 108 | 15 | 39 | 10 | 18.2%（10/55） | 96.9% (62/64) | 0 | 3.1% (2/64) | | 2.3 (1/43) | 32.6% (14/43) | 65.1% (28/43) |
| Spain^d^ | 15 | 11 | 4 | 1 | 3 | 100% (3/3) | 100% (7/7) | 0 | 0 | | nd^c^ | nd | nd |
| France^d^ | 140 | 94 | 18 | 28 | 9 | 60% (9/15) | 100% (84/84) | 0 | 0 | | 0 | 40.9% (18/44) | 59.1% (26/44) |
| US^d^ | 349 | 287 | 62 | nd | nd | nd | 100% (264/264) | 0 | nd | | 11.1 (7/63) | 88.9% (56/63) | nd |

^a^Genotypes include *erm(41)C28*, *erm(41)T28*, *M type* and *rrl* 2058/2059 mutation (*rrl* mut).

^b^Sensitive/ intermediate at ERT, but resistant at LRT.

^c^no data

^d^Data obtained from Brown-Elliott *et al.*, 2015; Rubio *et al.*, 2015 and Mougari *et al.*, 2016.
