# Supplementary material for: Relationship between Antibiotic Susceptibility and Genotype in Mycobacterium abscessus Clinical Isolates
Source: Front Microbiol. 2017 Sep 14;8:1739. doi: 10.3389/fmicb.2017.01739 (PMC5603792; doi:10.3389/fmicb.2017.01739)
Supplement: Supplementary file 2 [file Image1.pdf]

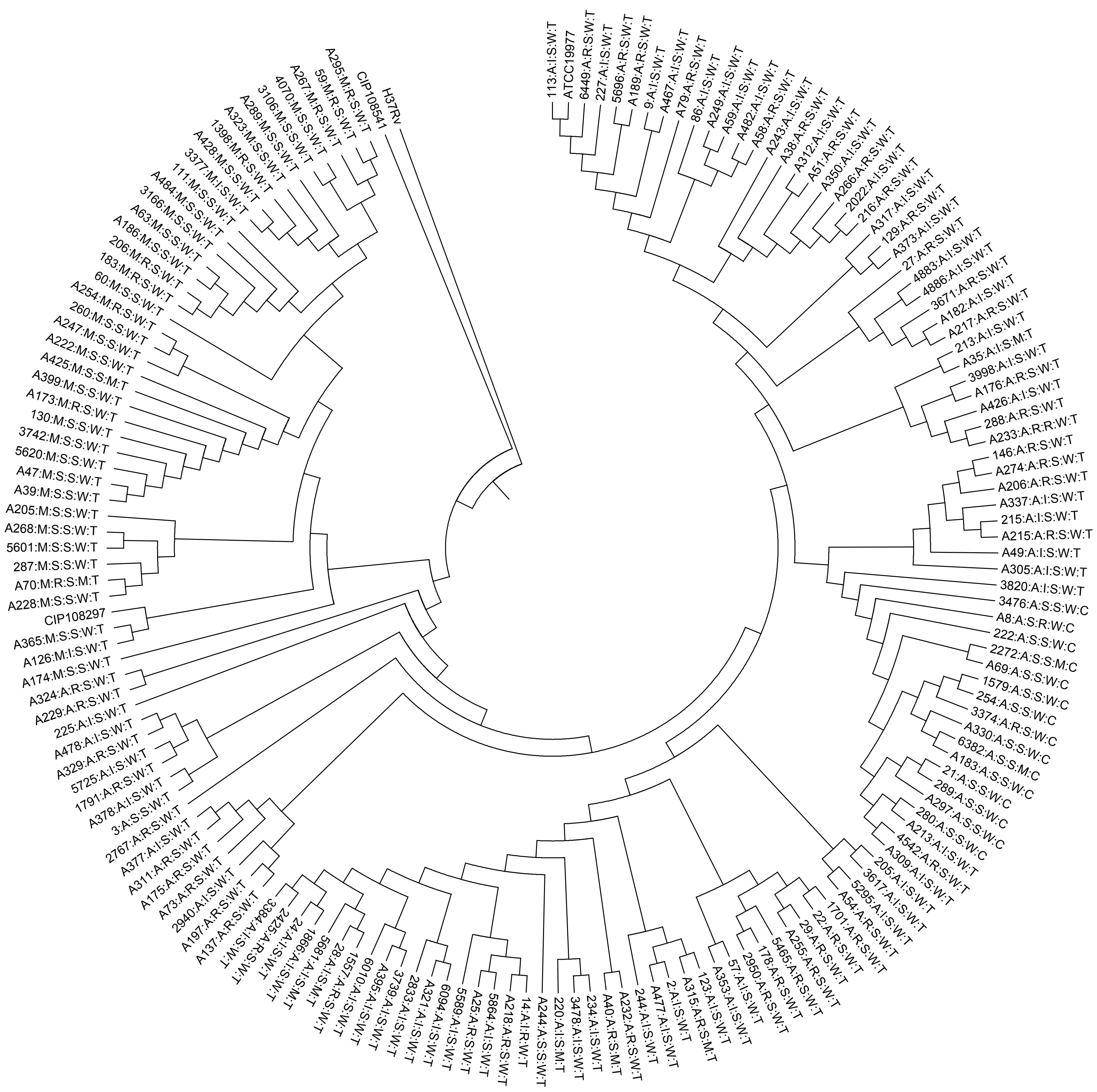

Supplementary Figure 1: Strain names were coded as follows, strain number: subtype (A/M): susceptibility of clarithromycin (S/I/R): susceptibility of amikacin (S/R): genotype of rrl (M/W): genotype of erm(41)(T/C). A, subsp. abscessus. M, subsp. massiliense. S, susceptible. I, inducible resistance. R, resistance. W, wile-type. M, mutant. T, erm(41)T28. C, erm(41)C28.
